# Immunopathogenesis in SARS-CoV-2 and *Mycobacterium tuberculosis:* The danger of overlapping crises

**DOI:** 10.3389/fphar.2022.1065124

**Published:** 2022-11-16

**Authors:** Prakasini Satapathy, Radha Kanta Ratho, Sunil Sethi

**Affiliations:** ^1^ Department of Virology, Postgraduate Institute of Medical Education and Research, Chandigarh, India; ^2^ Department of Medical Microbiology, Postgraduate Institute of Medical Education and Research, Chandigarh, India

**Keywords:** *Mycobacterium tuberculosis* (*M Tuberculosis*), COVID, coinfections, biomarkers (MESH D015415), treatment regimens

## Introduction

Severe acute respiratory syndrome coronavirus 2 (SARS-CoV-2), a member of the genus Betacoronavirus, was initially reported in Wuhan city, Hubei Province, China in late December 2019 (Gralinski and Menachery, 2020; Zhu et al., 2020). As SARS-CoV-2 spreads rapidly across the world, the World Health Organization (WHO) declared it a pandemic and public health emergency of international concern on 11 March 2020. The COVID-19 pandemic has severely impacted global public health activities, the economy, and curative services. It worsened the elimination program and adherence to treatment of TB, HIV (human immunodeficiency virus), malaria, measles, dengue fever, and neglected tropical diseases (NTDs) like lymphatic filariasis, soil-transmitted helminths, schistosomiasis, onchocerciasis, and trachoma (Mohan et al., 2021; Roberts, 2021; Toor et al., 2021; Aborode et al., 2022) As per 2021, WHO global survey report, 44% countries had disruption of NTD activities (World Health Organization (WHO), 2021a). Many ongoing NTD activities like mass administration campaigns of drugs and vaccines, case detection and vector control were postponed during the pandemic to avoid the additional transmission of SARS-COV-2 which ultimately leads to increased burden of NTDs in high transmission area (Toor et al., 2021).

TB is an infectious disease caused by *Mycobacterium tuberculosis*, is transmitted by aerosol affecting the lungs. It is a key public health concern due to mortality in low and middle-income countries. The majority of people exposed to MTB during childhood are asymptomatic and remain in latent form, whereas 5–10% of those exposed turn up with active disease (Dheda et al., 2017). National lockdown adversely affected TB care access, thereby leading to disease progression in many cases (Shariq et al., 2022). According to WHO, in 2020 death from *tuberculosis* increased from 1.4 million to 1.5 million with a 18% decline of new cases globally. Philippines (37%), Indonesia (31%), South Africa (26%) and India (25%) account for major declined of case detection globally (World Health Organization (WHO), 2021b). This requires further intervention to elucidate the risk factors in both SARS COV-2 and TB coinfection in terms of improvisation in case detection and management of TB in endemic countries.

## Clinical presentation of SARS COV-2 and MTB

Lungs are the common platform for both SARS-COV-2 and MTB, where both the pathogen replicate in alveolar macrophages and ciliated mucus-secreting epithelial type-2 pneumocytes. MTB utilises various pattern recognition receptors i.e., FCγ receptors, toll-like receptors, mannose receptors, complement receptors, nod- like receptors, dendritic cell-specific intercellular adhesion molecule grabbing nonintegrin, CD14 receptors, and scavenger receptors either singly or in combination (Russell, 2001). MTB induces the expression of ACE2 receptors for cell entry, which interestingly serves for the entry of SARS-COV-2 (Rosas Mejia et al., 2022), also thereby, sharing the common cell entry pathways. Patients with severe COVID-19 reported to have elevated levels of IL-2, IL-4, IL-6, IL-10, IFN-γ, TNF-α and G-CSF cytokines and chemokines in comparison to mild cases (Huang et al., 2020; Lee et al., 2021). Following alveolar entry, MTB infects type II pneumocytes, alveolar macrophages, and alveolar epithelial cells to release TNF-α, IL-1α. IL-1β, IFN-γ chemokines mediating inflammatory pathway (Etna et al., 2014). MTB favours intracellular survival through downgrading nitric oxide production, phagosomal maturation, and blocking IFN-γ signalling pathway in macrophages (Abdalla et al., 2016). Similarly Influenzae viruses also aggravate TB through elevated IL-10 in co-infected patients (Ring et al., 2019). There is every possibility that severe COVID-19 might reactivate the latent *tuberculosis* (LTBI) with in the patients.

T-cell-mediated immunity plays pivotal role in controlling disease progression. The frequencies of CD4 + T cells, CD8 + T cells, and NK cells reported to be low in COVID-19 patients associated with lymphopenia (Chen et al., 2020a; Tan et al., 2020), which possibly triggers the reactivation of LTBI (Amelio et al., 2019; Leonso et al., 2022). The mouse coronavirus model also reflects the reactivation of dormant TB by virus that triggering type-1 interferon signalling and activation of mesenchymal stem cell-based defence (Singh et al., 2020). Further studies have reported the reactivation of LTBI during corticosteroid (CST) therapy in COVID-19 patients, due to generalised immunosuppression (Gopalaswamy and Subbian, 2021; Friedman and DeGeorge, 2022).

## COVID-19 and tuberculosis coinfection

Table 1 shows a list of studies that reported coinfection of COVID-19 and TB and its severity. COVID-19 in TB patients is more commonly observed in high TB burden countries like India, China, and Vietnam (Dong et al., 2020). MTB infection in patients with COVID-19 was more commonly found than other comorbidities like diabetes, hypertension, and coronary heart disease (Guan et al., 2020). When comparing patients with TB and COVID-19 with pneumonia, 22% of the patients had mild clinical disease, while 78% of COVID-19 had increased severity (Chen et al., 2020b; Guan et al., 2020). Co-infection with SARS CoV-2 and MTB is of concern as the diagnosis of *tuberculosis* is more likely to be missed due to nonspecific presentation and a lack of typical radiological findings. Pre-existing TB and underlying lung comorbidities aggravate the disease in COVID-19 (Tadolini et al., 2020) possibly through alteration in metabolic pathways. A metabolomic analysis reveals low levels of metabolic biomarkers (Branch chain amino acids, Betaine and its derivatives) as a consequence of post TB infections, are associated with COVID-19 severity (Diboun et al., 2022).

**TABLE 1:** Studies reported COVID-19 and TB Coinfection.

Study	Country	COVID-19 and TB coinfection
Crowder et al. (2021) (Crowder et al., 2021)	Philippines	Two times higher risk with mortality and 25% lower recovery in COVID-19-TB co-infected patients in comparison to COVID-19 patients without TB. Further in Philippines there was an increase of 56.3% TB associated death due to health service disruption of TB care during COVID-19 pandemic.
Sereda et al. (2022) (Sereda et al., 2022)	Belarus	Reported 5.6% of active TB coinfection in hospitalised COVID-19 patients.
Boulle et al. (2022) (Boulle et al., 2021)	South Africa	South Africa with high TB and HIV burden has experienced surge of COVID-19 cases due to Omicron variant, 10% of COVID-19 patients of Western Cape Province had either history of TB or active TB (Boulle et al., 2021).
Kumar et al. (2021) (Kumar et al., 2021) Mathur et al. (2022) (Mathur et al., 2022)	India	Kerala reported 15.2% deaths in active TB-COVID-19 coinfection (Kumar et al., 2021), further a tertiary care hospital in India also showed association of TB (10%) in paediatric COVID-19 patients (Mathur et al., 2022).
TB/COVID-19 Global Study Group (2022) (TB/COVID-19 Global Study Group, 2022)	Multi-country study	A cohort study involving 34 countries reported, 12% mortality of coinfected patients associated with male and older age group (TB/COVID-19 Global Study Group, 2022).

Chen Y, et al., reported MTB and SARS-COV-2 coinfection induces disease progression and severity in hospitalized COVID-19 patients in China (Chen et al., 2020b). A modelling study by Hogan AB, et al., assumed that COVID-19 pandemic response could increase TB mortality up to 20% with in 2020 and 2025 (Hogan et al., 2020). The disruption epidemiological surveillance and reduction in *tuberculosis* tests due to COVID-19 pandemic might lead to increase in *tuberculosis* mortality. In addition to mortality treatment adherence and follow up of TB patients have been negatively affected.

Even though MTB is an apparent risk factor for COVID-19 aggravation, features like alcohol consumption, smoking, HIV and other viral, bacterial and fungal co-infections might have associated risk. Thus, clinical details and social determinants of coinfected patients needs to be assessed for the risk of morbidity and mortality. Early diagnosis of the disease or co-infections makes it mandatory for at risk and compromised patient groups for better management.

## Management of coinfection

Despite mass vaccination breakthrough COVID-19 infections have been reported in TB endemic countries, because of emergence of new variants of the SARS-CoV-2 that can escape the host’s immune response (Hacisuleyman et al., 2021; Prévost and Finzi, 2021; Cascella et al., 2022). A study demonstrated that in countries vaccinated with BCG, the frequency of the S 614G variant was associated with the highest mortality rate related to COVID-19 (Toyoshima et al., 2020).

According to the World Health Organization, exacerbation of TB appeared as the consequences of the COVID-19 epidemic. The possible key factors are: The emergence of COVID-19 pandemic has exerted high pressure on existing health system, weakened many national programmes including national TB elimination programme as well as the intricate association between the two pathogen within the host (Visca et al., 2021). This problem still needs a better evaluation of the coinfection of patients with TB and COVID-19.

Simultaneous testing for TB and COVID-19 may help in detecting new TB cases that missed public services in the context of COVID-19 (MacLean et al., 2022). Some of the strategies adopted to control COVID-19 pandemic may be implemented towards strengthening TB control programme like, teleconsultation, virtually support for self-administration of therapy to avoid delay in treatment, contact tracing and community awareness about any changes in health services etc. Hotspot mapping for active cases could help to identify the undiagnosed TB cases during the pandemic. Further social distancing to be implemented with MDR TB patients living overcrowded location with poor sanitation.

## Way forward

The rapid spread of the new variants of SARS-CoV-2 and drug resistance MTB has warned the public health system and requires active molecular and genomic surveillance of disease transmission and pathogenicity. It is important to recuperate in massive screening, case finding, including targeting high risk groups and allocation of more resources to find the missed TB cases during the COVID-19 pandemic to achieve the end goal of TB. Other chronic diseases, especially those spread through close contacts, should not be ignored in pandemic times and utmost care must be taken to avoid mortality from coinfection and inaccessibility of timely treatment.

## Abbreviations

ACE, Angiotensin-converting enzyme; CD, Clusters of differentiation; FC receptor, Fragment crystallizable receptor; G-CSF, Filgrastim and granulocyte colony-stimulating factor; IL, Interleukin; INF, Interferons; NK cells, Natural Killer cells; SARS-CoV-2, Severe acute respiratory syndrome coronavirus 2; T cells, T lymphocytes; TNF, Tumor necrosis factor.
